# Combined endoscopic surgery in the prone-split leg position for successful single-session removal of an encrusted ureteral stent: a case report

**DOI:** 10.1186/1752-1947-8-128

**Published:** 2014-04-17

**Authors:** Tetsuya Isero, Shuzo Hamamoto, Satoshi Koiwa, Hiroyuki Kamiya, Yoshihiro Hashimoto, Takahiro Yasui, Yutaka Iwase, Kenjiro Kohri

**Affiliations:** 1 Department of Urology, Toyota Kosei Hospital, 500-1 Ibohara, Jyousui-cho, Toyota City, Aichi 470-0396, Japan; 2 Department of Nephro-urology, Nagoya City University Graduate School of Medical Sciences, 1 Kawasumi, Mizuho-cho, Mizuho-ku, Nagoya 467-8601, Japan

**Keywords:** Encrusted stent, Combined endoscopic surgery, Prone split-leg position

## Abstract

**Introduction:**

Although encrusted stents may lead to some unwanted complications including urinary tract obstruction, urinary sepsis, and potential loss of kidney function, there is currently no consensus on the most efficient method for managing stents that are intentionally left *in situ*. This is the first report describing the management of an encrusted stent using combined endoscopic surgery in the prone split-leg position in a single session.

**Case presentation:**

A 47-year-old Asian man presented with left flank pain and macrohematuria. The patient had undergone left ureteral stenting three years previously for the treatment of left ureteral stones and hydronephrosis; however, he was lost to follow-up before the treatment for the ureter stones was completed. Therefore, the ureteral stent and stones were not removed. An abdominal radiograph and a noncontrast computed tomography scan showed encrustation along the retained stent with stone burdens in the kidney and ureter. The ureteral stent could not be removed by cystoscopy after shock wave lithotripsy of the left ureteral stones. Therefore, endoscopic lithotripsy combined with flexible ureteroscopy and miniature nephroscopy was performed with the patient in the prone split-leg position. All the stones and the encrusted ureteral stent were successfully removed in a single session.

**Conclusions:**

In this case, percutaneous nephrolithomy in addition to flexible ureteroscopy was preferred because severe encrustation of the proximal stent and ureteral stones complicated the therapeutic strategy. Combined endoscopic techniques in the prone split-leg position can achieve successful and safe management of encrusted stents.

## Introduction

In 1967, Zimskind *et al.* first reported the use of silicone ureteral splints to relieve ureteral obstructions [1]. The use of ureteral stents has become routine in urological procedures, including the treatment of obstructing ureteral calculi, ureteral strictures, ureteropelvic junction obstructions, or after open or endoscopic ureteral surgery. However, ureteral stent placement may lead to some unwanted adverse effects and complications, including migration, fragmentation, and stone formation; furthermore, the stent removal may be overlooked. The initial recommendations for management of an encrusted stent include shock wave lithotomy (SWL) and ureteroscopy (URS); however, many problems are associated with complete removal, including a long treatment duration, complicated technique, and high costs. This report presents a case of successful removal of an encrusted ureteral stent in a single session by combined endoscopic surgery using miniature percutaneous nephrolithotripsy (mini-PNL) and flexible URS (fURS).

## Case presentation

In September 2009, a 47-year-old Asian man underwent ureteral stenting of the left ureter for the treatment of left ureter stones and hydronephrosis; however, he was lost to follow-up before complete removal of the stones and stent. He was referred to our department because of a complaint of left flank pain. His medical history included hepatitis C and fracture of the right femur. There was no related family history.

**Figure 1:**
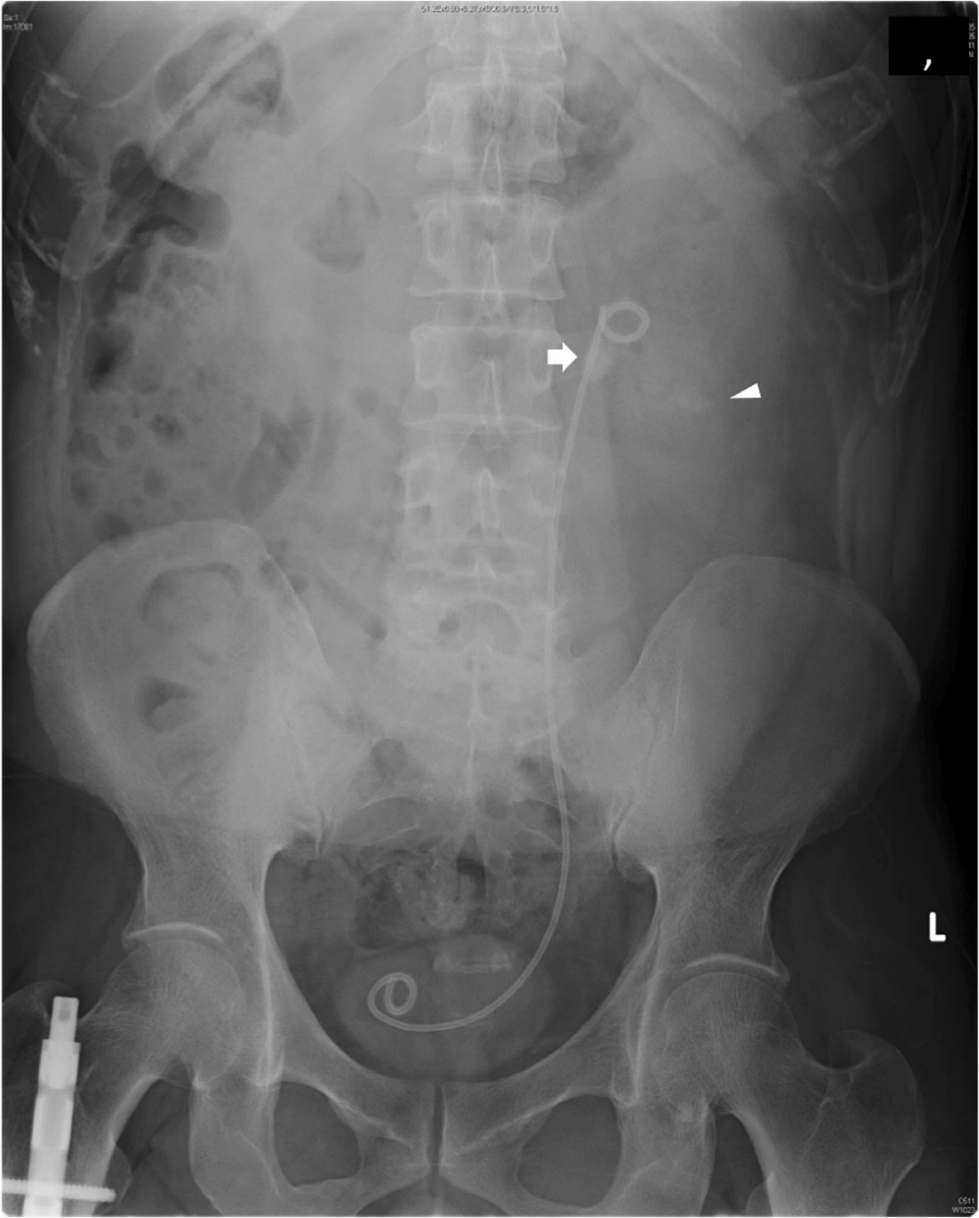
**The kidney, ureter and bladder** (**KUB) X-ray shows a retained stent with stone burden in the ureter and kidney.** The arrow indicates ureteral stones. The arrowhead indicates renal stones.

A physical examination revealed only left costovertebral angle tenderness and no other abnormal physical findings. An abdominal radiograph (Figure 1) and a computed tomography (CT) scan were obtained. An abdominal radiograph showed stones in the left ureter and kidney and encrustation along the ureteral stent. A CT scan revealed that the encrustation covered the entire stent, resulting in left hydronephrosis. A laboratory examination showed no abnormalities. Urinalysis showed a red blood cell count of >100/hpf and a white blood cell count of >30 to 49/hpf. A urinary culture showed the presence of *Escherichia coli*.

**Figure 2:**
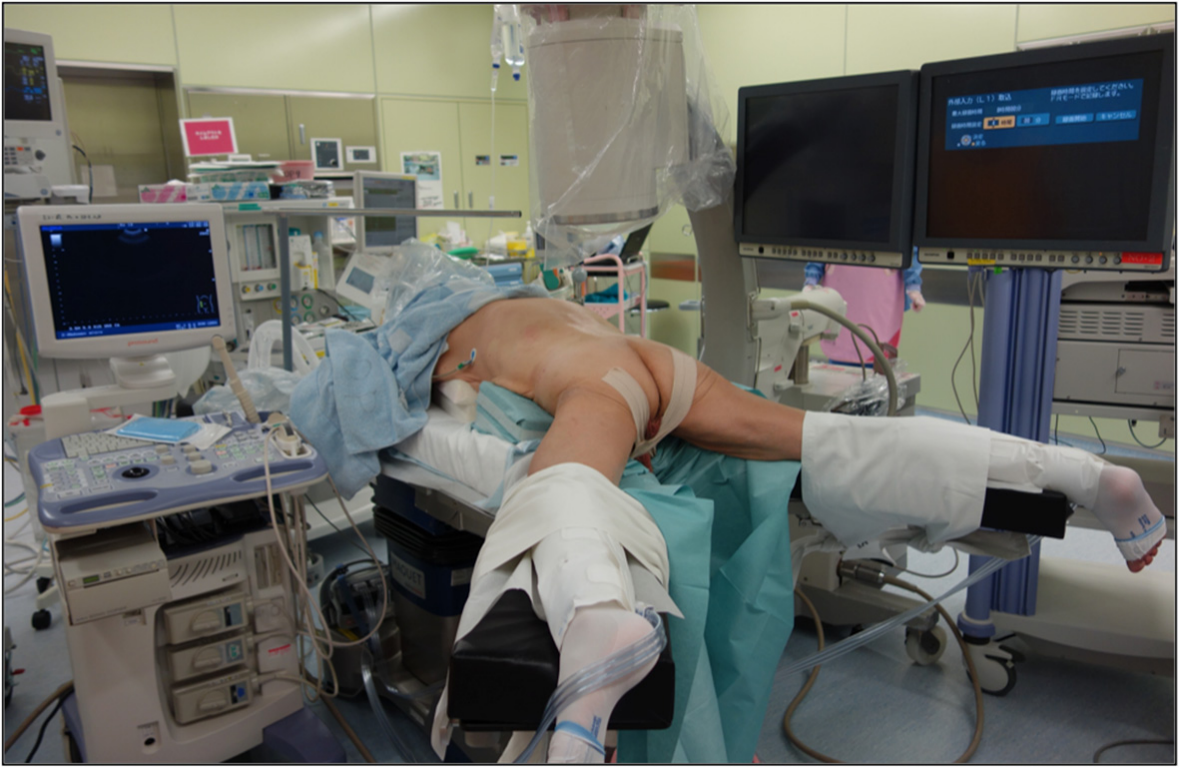
Patient positioning in the prone split-leg position.

**Figure 3:**
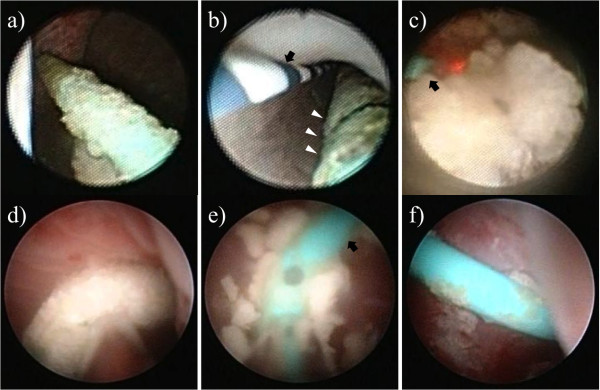
**Ureteroscopic (a-c) and nephroscopic images (d-f). (a)** The distal end of the stent was heavily encrusted. **(b)** A ureteroscope was inserted beside the encrusted ureteral stent in the direction of the ureteral stones at the upper ureteral tract (arrow: 0.035-mm guide wire, arrowheads: encrusted stent). **(c)** Lithotripsy of the ureteral stones using a Holmium YAG laser (arrow: laser fiber). **(d)** The proximal end of the encrusted stent. **(e)** Lithotripsy of the encrusted stent using lithoclast (arrow: ureteral stent). **(f)** Ureteral stent after fragmentation of the encrustation.

**Figure 4:**
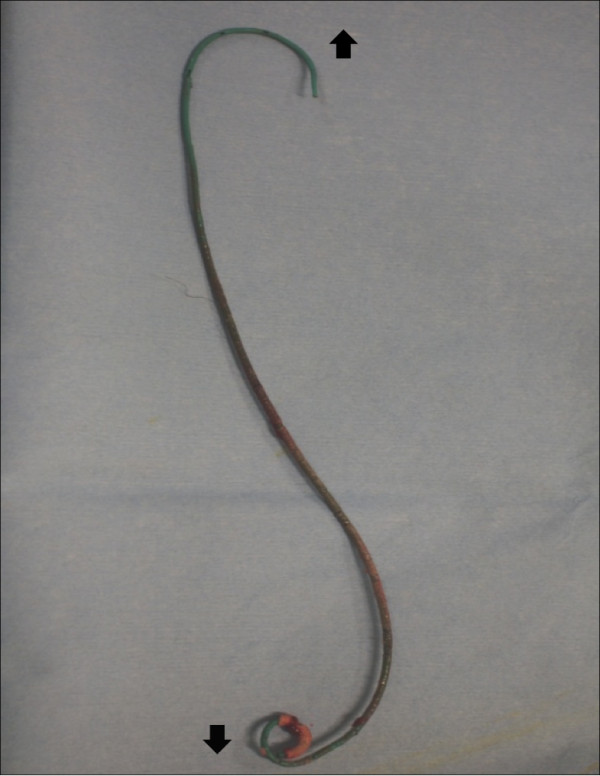
**The retrieved stent.** [↑] indicates the proximal side of the ureteral stent. [↓] indicates the distal side of the ureteral stent.

One week later, the patient was admitted, and an attempt to remove the stent by cystoscopy after SWL was unsuccessful. One week later, under general anesthesia, stent removal was attempted by combined endoscopic surgery using fURS and mini-PNL. The patient was oriented in the prone split-leg position throughout the operation, allowing both retrograde and antegrade access (Figure 2). The procedure was performed by two urologists working simultaneously to fragment the renal stones; one performed fURS (Figure 3a-d), and the other performed mini-PNL (Figure 3e-h). Flexible cystoscopy was performed to observe the stent encrustation and locate the ureteral orifice. The distal end of the ureteral stent was highly encrusted (Figure 3a). Under fluoroscopic guidance, the ureteral orifice was cannulated with a 0.035-mm guide wire that was passed into the upper urinary tract, and a ureteroscope (Flex X-2™, Karl Storz, Tuttlingen, Germany) was inserted beside the encrusted stent toward the ureteral stones in the upper ureteral tract (Figure 3b). The ureteral stones and the encrustation were fragmented using a Holmium-yttrium aluminum garnet (YAG) laser (a 200-μm fiber 1.5Hz 8H; VersaPulse^®^ 80W, Lumenis Inc, San Jose CA, USA) (Figure 3c). The stent could not be removed successfully after retrograde lithotripsy with fURS, because of severe proximal encrustation of the stent. Renal puncture was achieved using ultrasonography under fluoroscopic guidance. An 18-Fr mini-PNL tract (Karl Storz) was used to dilate the tract and establish working access. To fragment the proximal encrustation and renal stones, lithoclast lithotripsy (Boston Scientific Japan, Tokyo, Japan) was performed using a 12-Fr mini-nephroscope (Karl Storz) (Figure 3d-f). Stones were broken into smaller fragments and washed through the sheath by retrograde irrigation. After fragmentation of both ends of the encrustation, the stent was removed by cystoscopy (Figure 4). The urinary tract was stented with a 4.7-Fr double-J ureteral stent and an 18-Fr nephrostomy tube. The total operation time was 124 minutes. The nephrostomy tube was removed two days after surgery. The ureteral stent was removed one month later. An analysis of the encrusting material showed the presence of calcium-oxalate and calcium-phosphate calculi.

## Discussion

Since the introduction of ureteral stents in 1967, they have become essential in all aspects of urology, including the management of ureteral obstruction due to intrinsic or extrinsic causes, or after open or endoscopic ureteral surgery [1-3]. Various materials and coatings have been developed for stents; however, serious complications, including encrustation, fragmentation, migration, and infections still occur, especially when stents have been left *in situ* for a long time [4].

Stent encrustation is caused by the precipitation of uric acid or calcium oxalate onto the surface of the stent [5]. Moreover, severe encrustation with stone formation can lead to urinary tract obstruction, urinary sepsis, and potential loss of kidney function [6]. Ureteral stent encrustation is related to the indwelling duration. Some reports have documented that the stent encrustation rate increases from 9.2% to 26.8% for a stent placement duration of <6 weeks, to 47.5 to 56.9% at 6 to 12 weeks, and 76.3 to 75.9% at >12 weeks [7]. Therefore, a ureteral stent requires occasional replacement, and indwelling duration should be minimized to avoid complications.

Stent encrustation can pose a serious challenge to urologists, and currently, there is no consensus regarding the most efficient method for managing stents that are unintentionally left *in situ*. SWL has been proposed by several studies because it is less invasive. URS in combination with a holmium laser lithotripter represents another minimally invasive management option. However, PNL in conjunction with antegrade URS is the preferred option for severe encrustation of a proximal stent [8]. Moreover, PNL facilitates concomitant removal of renal stone fragments. In contrast, Bultitude reported that endoscopic SWL and URS, either alone or in combination, are recommended as first-line treatments, and that resorting to PNL should only be considered if these options fail [9]. Weedin reported that distal stone burden can usually be treated by procedures involving less morbidity, such as cystolithotripsy and/or rigid URS, whereas proximal encrustation might require fURS or PNL [8].

A major consideration in the management of an encrusted stent is to minimize the number of interventions needed to achieve stone-free and stent-free status. The number of endourologic procedures reported to achieve this ranges from 2.7 to 4.2 [10]. A novel technique using PNL combined with retrograde fURS for treating large kidney stones (endoscopic combined intrarenal surgery; ECIRS) was developed to decrease the number of percutaneous tracts and yield a one-step procedure with a high stone-free rate [11-14]. The simultaneous antero-retrograde approach decreases absorption of the irrigation fluid into the circulation, and affords better visibility because of the use of ureteroscopic and nephroscopic irrigation. After removal of almost all the stones, residual stones can be located using fURS. In the present case, ureteral stones and the associated encrustations were treated using fURS; however, the stent could not be removed after the fURS lithotripsy because of the proximal encrustation. For the management of severe proximal encrustation of the stent, PNL facilitated direct access to the renal pelvis. Renal stones and the proximal encrustation of the stent were treated using mini-PNL and all urinary stones and the encrusted stent were successfully removed in a single session.

Combination of endourological procedures into a single session commonly requires intraoperative repositioning; URS is usually performed in the dorsal lithotomy position, and PNL is usually performed in the prone position. In 1988, Bagley and Lehmanand used a modified prone position for nephroscopic and ureteroscopic procedures in female patients [11]. Five years later, a prone split-leg position was reported with simultaneous anterograde and retrograde endoscopy using a two-team approach [12]. However, recently, the Galdakao-modified supine Valdivia position is being adopted more frequently for the simultaneous use of fURS and PNL [13]. The supine position provides some advantages, as it requires less time for patient positioning, results in lower pressure on the renal pelvis (reducing the risk of fluid absorption), and enables ureteroscopic access [13]. It is also associated with a lower risk of cardiovascular, respiratory, neuroendocrine, and pharmacokinetic anesthesia problems that are typically observed when using the prone position, particularly in obese patients [15]. However, the supine position has certain disadvantages such as constant collapse of the pyelocaliceal system and the small range of potential access angles, which may increase the risk of visceral injury [16]. In the modified Valdivia position, the kidney is hypermobile, which may increase the risk of renal puncture and makes guidewire manipulation more difficult. This leads to longer tracts and reduced nephroscope mobility, especially in obese patients. Therefore, a greater torque is required to manipulate the scope, which consequently increases the possibility of damage to the renal parenchyma and bleeding from the tract [17].

The combined procedure described here was performed with the patient in the prone split-leg position and has distinct advantages. First, there is no need for intraoperative repositioning of the anesthetized patient. Second, the prone position for PNL is frequently used by urologists, and it provides a larger area for percutaneous renal access and allows a wider space for instrument manipulation. Third, the prone position is also associated with a significantly lesser nephrostomy tract length and a greater number of potential access sites, which may improve the ease and safety of PNL [14,17].

## Conclusions

To the best of the authors’ knowledge, this is the first reported case of the management of an encrusted stent using combined endoscopic surgery in the prone split-leg position in a single session. Thus, we noted that combined endoscopic techniques in the prone split-leg position could achieve successful and safe management of encrusted stents.

## Consent

Written informed consent was obtained from the patient for publication of this manuscript and any accompanying images. A copy of the written consent is available for review by the Editor-in-Chief of this journal.

## Abbreviations

CT: Computed tomography; ECIRS: Endoscopic combined intrarenal surgery; fURS: flexible URS; mini-PNL: miniature percutaneous nephrolithotripsy; SWL: Shock wave lithotomy; URS: Ureteroscopy.

## Competing interests

The authors declare that they have no competing interests.

## Authors’ contributions

SH was a major contributor in the writing of this manuscript. TI, SH, SK, and HK performed the surgery and TI and SH analyzed the data. SH, YH, TY, YI, and KK provided important intellectual content and helped revise the manuscript. All authors read and approved the final manuscript.
